# Association of Serum Alpha-Tocopherol and Retinol with the Extent of Coronary Lesions in Coronary Artery Disease

**DOI:** 10.1155/2018/6104169

**Published:** 2018-12-11

**Authors:** Carolinne Thaísa de Oliveira Fernandes Miranda, Victor Hugo Rezende Duarte, Marina Sampaio de Menezes Cruz, Mychelle Kytchia Rodrigues Nunes Duarte, Jéssica Nayara Góes de Araújo, Ayda Maria Quirino Silva dos Santos, Juliana Marinho de Oliveira, Maria Sanali Moura Oliveira Paiva, Adriana Augusto Rezende, Mario Hiroyuki Hirata, Rosario Dominguez Crespo Hirata, Karla Danielly da Silva Ribeiro, André Ducati Luchessi, Vivian Nogueira Silbiger

**Affiliations:** ^1^Laboratory of Bioanalysis and Molecular Biotechnology, Department of Clinical and Toxicological Analyses, Federal University of Rio Grande do Norte, Natal, Brazil; ^2^Department Cardiology, Onofre Lopes University Hospital, Natal, Brazil; ^3^Multidisciplinary Laboratory, Department of Clinical and Toxicological Analyses, Federal University of Rio Grande do Norte, Natal, Brazil; ^4^Department of Clinical and Toxicological Analyses, School of Pharmaceutical Sciences, University of Sao Paulo, São Paulo, Brazil; ^5^Department of Nutrition, Federal University of Rio Grande do Norte, Natal, Brazil

## Abstract

*Background and aims*: Fat-soluble vitamins play an important role in the pathogenesis of cardiovascular disease and progression of atherosclerosis. This study aimed at investigating the relationship of the serum levels of alpha-tocopherol and retinol with the extent of coronary lesions in patients with coronary artery disease. *Methods*. Patients with coronary artery disease (*n*=177) aged 30–74 years, who underwent their first coronary angiography, were enrolled. The extent of coronary lesions was assessed using the Friesinger index (FI). Accordingly, patients were grouped as follows: FI = 0–4 (*n*=90), FI = 5–9 (*n*=50), and FI = 10–15 (*n*=37). Serum levels of vitamins were ‬determined via high-performance liquid chromatography and serum biochemical analysis. *Results*. Assessment of FI-based groups revealed that 50.8% patients had a coronary artery lesion to a low extent (FI 0–4). Individuals in this group were younger and had lower glucose and serum alpha-tocopherol levels than the other groups (*p* < 0.05). Low levels of alpha-tocopherol were more frequent in the FI 0–4 group than that in the other groups (*p*=0.03). No difference was observed between the mean serum retinol levels among the FI-based groups (*n*=0.492), and the low frequency of retinol was consistent among the FI groups (*n*=0.348). *Conclusions*. The low level of alpha-tocopherol together with the presence of dyslipidemia is probably associated with the initial events in atherosclerosis. Increased alpha-tocopherol levels in patients with more extensive coronary artery lesions may have resulted from altered vitamin E metabolism with increased oxidative stress.

## 1. Introduction

Coronary artery disease (CAD) is the leading cause of mortality among humans, with an estimated worldwide mortality rate of 32% [1]. CAD is a chronic disease caused by atherosclerosis. Often, the early stage of the disease is asymptomatic, and the diseases progresses gradually [2, 3]. Atherosclerosis is a chronic inflammatory vascular disease and major cause of cardiovascular diseases (CVD) [4].

CAD results from the formation of atherosclerosis plaques and is the primary lesion in the disease. Atherogenesis may lead to endothelial injury, owing to the oxidative stress and inflammation induced by the exposure to metabolic alterations and environmental factors including tobacco smoking, type 2 diabetes, hypertension, dyslipidemia, and obesity [5]. Management of these metabolic diseases may decelerate CVD progression.

Fat-soluble vitamins may prevent oxidative stress, primarily by reducing low-density lipoprotein (LDL) peroxidation [6]. Serum levels of antioxidant vitamins such as vitamin E (alpha-tocopherol) and vitamin A (retinol) are seemingly important in reducing the risk of CAD, thereby playing a potentially important role in reducing the risk of CVD and deterring atherogenesis [7–9].

Alpha-tocopherol is a common form of vitamin E with the highest antioxidant activity. It is the preferentially absorbed and accumulated form of vitamin E [10]. Alpha-tocopherol levels are inversely proportional to CAD occurrence [6, 9]. Vitamin E regulates proteins involved in the uptake, transport, and degradation of atherogenic lipids and displays anti-inflammatory activity [11, 12].

Vitamin A is also important for maintaining immune function and plays an essential role in the development of T-helper and B cells [13]. The primary forms of vitamin A are retinol, retinoic acid, and retinal, whereas the form stored in the liver is retinyl palmitate [14]. Retinol is one of the circulating forms of plasma vitamin A. Low retinol levels are reportedly a characteristic of unstable atherosclerotic plaques [15]. Patients with CAD or atherosclerosis may have significantly lower circulating levels of retinol [16]. Reduced retinol has been suggested to be a risk factor for CVD and mortality [7, 15, 17].

Nutrition is one of the most critical factors influencing the CVD pathogenesis. Thus, monitoring of vitamin levels in biological fluids is a useful method to determine the nutritional status of patients. However, the association between fat-soluble antioxidant vitamins and the extent of coronary lesions is unclear.

This study aimed to examine the association of serum concentrations of alpha-tocopherol and retinol with the extent of coronary lesions in individuals diagnosed with CAD via coronary angiography.

## 2. Materials and Methods

### 2.1. Study Population

A total of 177 subjects (30–74 years old), who underwent their first coronary angiography for CAD diagnosis, were enrolled in this study, as described previously [18, 19]. Patients were selected at the hemodynamics unit of the Hospital Universitário Onofre Lopes, Instituto do Coração, and Natal Hospital Center in Natal and Rio Grande do Norte State in Northeast Brazil. The study was approved by the hospital's Research Ethics Committee (CAAE 0001.0.051.294-11). All participants provided written informed consent, and the study protocol conformed to the ethical guidelines of the 1975 Declaration of Helsinki.

Information regarding age, body mass index, sex, hypertension, obesity, cigarette smoking, physical activity, alcohol consumption, and a family history of CAD was recorded. Exclusion criteria were a diagnosis of cardiomyopathy, heart valve disease, congenital diseases, pericarditis, chronic kidney disease, liver failure, endocrine disorder (except type 2 diabetes), inflammatory diseases, malignant diseases, blood disorders, autoimmune diseases, family history of hypercholesterolemia, and previous cardiovascular events, including acute coronary syndrome or coronary revascularization.

The extent of coronary artery lesion was assessed using the Friesinger index (FI) [18, 20]. Each of the three primary coronary arteries (anterior descending, circumflex, and right coronary) was scored separately from zero to five, within a range of 0–15. All coronary lesions were assessed by the same group of interventional cardiologists. In accordance with Chagas et al. [20], patients were grouped as follows: FI = 0–4, FI = 5–9, and FI = 10–15 [20].

### 2.2. Blood Sampling and Laboratory Analysis

Peripheral blood samples were drawn from patients before coronary angiography in tubes without anticoagulant for biochemical analysis, as described previously [18]. Fasting serum glucose, triglycerides, total cholesterol, high-density lipoprotein cholesterol, urea, creatinine, uric acid, and alanine and aspartate aminotransferase levels were measured via colorimetric and kinetic ultraviolet methods using a Labmax Plenno biochemical analyzer (Labtest, Minas Gerais, Brazil). Values of LDL cholesterol were calculated in accordance with the Friedewald formula [21].

### 2.3. Quantification of Serum Alpha-Tocopherol and Retinol Levels

Alpha-tocopherol and retinol ‬were ‬extracted ‬‬from ‬serum samples via our previously reported method with slight modification [22, 23]. In brief, ‬95% ethanol ‬and hexane ‬P.A (Merck, ‬Darmstadt, Germany) were ‬used ‬for ‬protein ‬precipitation ‬and ‬for ‬extraction, respectively. ‬After evaporation ‬under a ‬nitrogen ‬atmosphere, ‬the ‬extract ‬was diluted ‬in ‬‬absolute ‬ethanol ‬(Vetec, ‬St. ‬Louis, MO, USA).

Serum alpha-tocopherol and retinol levels were determined via high-performance liquid chromatography (HPLC) analysis, using an HPLC device (Shimadzu, Kyoto, Japan) and a reverse-phase C18 column (LiChroCART 250–4; Merck, Darmstadt, Germany) coupled with an SPD-20A UV-Vis detector and LC Solution software (Shimadzu) for data processing. Twenty microliter (20 *μ*L) extracts were used for HPLC analyses. The mobile phase was 100% methanol in an isocratic system with a flow rate of 1.0 mL/min and the detector wavelength adjusted to 292 nm for alpha-tocopherol and to 325 nm for retinol.

Alpha-tocopherol and retinol levels were quantified via area under curve analysis, using respective standards as references (Sigma-Aldrich, St. Louis, MO, USA). Concentrations of the standards were confirmed from the specific extinction coefficients of alpha-tocopherol (1%, 1 cm = 75.8) [24] and retinol (1%, 1 cm = 1780) [25] in absolute ethanol (Merck). These standards were used to generate the calibration curves. The linearity of the HPLC method ranged from 3.4 to 53.7 *μ*mol/L and from 3.2 to 102 *μ*mol/L (*r* = 0.9998) for alpha-tocopherol and retinol, respectively.

Serum concentrations of alpha-tocopherol and retinol >12 *μ*mol/L and 0.70 *μ*mol/L, respectively, were considered adequate [26, 27].

### 2.4. Statistical Analysis

Statistical analyses were performed using SPSS 22.0 software (SPSS, Chicago, IL, USA). Normally distributed data were evaluated using the Kolmogorov–Smirnov test. Normally distributed continuous variables are presented as mean and standard deviation values and were compared using ANOVA followed by Tukey's test for post hoc analysis. Variables with skewed distributions are presented as the median and interquartile range and analyzed using the Kruskal–Wallis test followed by Mann–Whitney test. Categorical variables are presented as the number of subjects and percentages and were compared using the chi-square test. Independent variables possibly affecting FI were determined by a model of multivariate regression analysis. A *p* value <0.05 was considered statistically significant.

To assess the association between alpha-tocopherol and retinol levels with coronary lesion extensions in patients with CAD, the required sample size was estimated to be 84 for a three-tailed significance level of 95%, power of 0.8, and average effect of 0.35 (software G^*∗*^POWER, version 3.1.9.2, University of Düsseldorf, Düsseldorf, Germany) [28].

## 3. Results

Clinical and laboratory data of CAD patients are enlisted in Table 1. Assessment of FI groups revealed that 50.8% (*n*=90) patients had coronary artery lesions to a low extent (FI 0–4). Subjects in this group were younger and displayed significantly lower glucose levels than those with higher FI (*p* < 0.05). Clinical and other laboratory variables were similar among FI groups (*p* > 0.05).

Serum alpha-tocopherol and retinol levels were analyzed in 82 and 141 CAD patients, respectively. The mean alpha-tocopherol level was lower in the FI 0–4 group than in the FI 5–9 group (*p* < 0.05) (Table 2). Low alpha-tocopherol levels were significantly more frequent in the FI 0–4 group (33.3%) than in the FI 5–9 (13.0%) and FI 10–15 (5.9%) groups (*p*=0.033). In contrast, mean serum retinol levels remained largely unchanged among the FI groups, and the frequency of low retinol levels was similar among FI groups.

Multiple logistic regression showed that variables age and alpha-tocopherol were independently associated with FI (Table 3). Patients with alpha-tocopherol values >12 *μ*mol/L had greater likelihood of having FI 10–15 (OR: 11.5, 95% CI = 1.182–112.058, *p*=0.035).

## 4. Discussion

Fat-soluble antioxidant vitamins have long been associated with a decreased risk of CAD [6]. Thus, in the present study, we evaluated the association of serum alpha-tocopherol and retinol levels with the presence and extent of coronary lesions. Among clinical and biochemical parameters of patients, the characteristics of the subjects were consistent with the factors associated with the disease, such as obesity, dyslipidemia, diabetes, and hypertension, among other analyzed variables displayed in Table 1.

To our knowledge, this is the first study to assess the plausible association between circulating tocopherol and retinol levels with the extent of coronary lesions in CAD patients. This study primarily shows that serum alpha-tocopherol levels were lower in the FI 0–4 group than in the FI 5–9 group and that a low alpha-tocopherol level was more frequent in the FI 0–4 group. A low alpha-tocopherol level together with the presence of dyslipidemia suggests that the CAD patients in the FI 0–4 group had reduced antioxidant protection by alpha-tocopherol and hence may have been more prone to the induction of initial events of the atherosclerotic process.

Tocopherols as antioxidants may be promising agents in reducing the risk of CAD. This is based on the hypothesis that inhibits LDL oxidation, thereby protecting against atherogenesis. In addition, tocopherols inhibit platelet aggregation and thrombogenesis [29]. The present findings are concurrent with previous reports that vitamin E deficiency manifests in the early stage of atherosclerosis [30].

Recent studies have reported that dietary antioxidants are important in preventing chronic diseases. For example, adequate intake of different dietary antioxidants, such as vitamin E and vitamin A, is associated with a lower risk of chronic diseases [31]. We acknowledge the importance of dietary evaluation in the present study. However, this was not essential in the present analysis because dietary ingestion is only a determining factor in cases of supplementation, which did not occur in the present study. Moreover, measures of dietary intake, irrespective of accuracy, do not reflect the bioavailability of the nutrients from various food items, the level of absorption in the digestive tract, or individual metabolic differences. Nutritional biomarkers reflect habitual intake or a steady state of intake and metabolism [32].

The use of a dietary questionnaire may represent external exposure, whereas blood biomarker analysis better reflects the internal state [33]. Plasma alpha-tocopherol concentrations may represent the most relevant biological exposure in comparison with intake estimates [34]. Tocopherols are more rigidly regulated by homeostatic systems and are therefore less reflective of food intake. A study reported that plasma alpha-tocopherol concentrations measured via NHANES III were not correlated with estimated vitamin E intake from 24 h dietary recalls [35]. Another study also reported that plasma concentrations of alpha-tocopherol were not associated with intake estimates or that the observed associations largely resulted from supplemental intake of vitamin E [26]. In addition, no studies have reported that vitamin intake does not determine the current alpha-tocopherol levels; these levels are rather affected by the levels of circulating lipids [36]. Moreover, only retinol levels are altered in response to extreme vitamin A deficiency or disease states [37] and in the postprandial period when a vitamin A-rich meal is provided to individuals with vitamin A deficiency [38].

In the present study, no differences were observed in serum lipids among the FI groups. However, serum alpha-tocopherol levels were highly dependent on levels of circulating lipids. Tocopherols are transported in LDL particles to peripheral tissues. Elevated LDL levels are associated with an increase in the levels of these antioxidants [39, 40], thereby potentially explaining why levels of alpha-tocopherol were higher in the FI 5–9 and FI 10–15 groups. In addition, these data were also confirmed by multivariate regression analysis.

In addition, alpha-tocopherol transfer protein (*α*-TTP) is required for trafficking to allow the liver to export alpha-tocopherol to the plasma. Oxidative stress may upregulate *α*-TTP transcription [40]. Our findings are consistent with a report [40] regarding increased transport of alpha-tocopherol from the liver to the plasma in response to oxidative stress via *α*-TTP upregulation.

The inconsistent findings regarding serum tocopherol levels and CVD may reflect a paradoxical effect of tocopherol on oxidation. Alpha-tocopherol has neutral, anti-, or even prooxidant activity under various conditions [41, 42].

Although there are no studies on the relationship between retinol levels with FI, low plasma retinol was found in individuals with atherosclerosis. In addition, the unstable atherosclerotic plaque is also characterized by low retinol content [7, 15, 43]. Our results corroborate these findings; however, we do not find statistically significant difference in the mean serum retinol levels among FI groups.

Our study has some limitations of note. The diet of CAD patients and oxidized LDL levels were not analyzed, and the study involved a small cohort. Although the cohort size was large enough to permit statistical analysis of the association between vitamins and the extent of coronary lesions, future studies with larger cohorts with a greater number of individuals in each FI group are required to improve the statistical power and validity of our results.

## 5. Conclusion

Although a low level of alpha-tocopherol together with the presence of dyslipidemia is probably associated with an initially higher CAD risk, this hypothesis is more speculative, as there is no group of age-matched subjects without CAD for comparison. In addition, the increased levels of alpha-tocopherol in patients with more extensive coronary artery lesions may reflect changes in vitamin E metabolism in situations involving greater oxidative stress. Circulating alpha-tocopherol alone does not protect against CAD pathogenesis. Further prospective studies are required for validation and to determine whether monitoring of alpha-tocopherol levels would be valuable for the assessment of the risk of CVD.

## Figures and Tables

**Table 1:** Demographic, anthropometric, and clinical data of patients with coronary artery disease.

Variables	Total (*n*=177)	Extent of coronary lesion (Friesinger index)			*p* value
		0–4 (*n*=90)	5–9 (*n*=50)	10–15 (*n*=37)	
Age (years)	58 ± 10	56 ± 10	60 ± 11	62 ± 7	0.004^a^
Men, *n* (%)	99 (55.9)	48 (53.3)	30 (60.0)	21 (56.8)	0.592
BMI (kg/m^2^)	27.8 ± 54.9	28.3 ± 5.1	26.8 ± 4.5	28.1 ± 4.5	0.212
Obesity, *n* (%)	45 (27.1)	17 (25.4)	8 (18.6)	4 (12.9)	0.279
Dyslipidemia, *n* (%)	165 (93.2)	82 (91.1)	48 (96.0)	35 (94.6)	0.508
Type 2 diabetes, *n* (%)	49 (27.7)	20 (24.4)	17 (34.0)	12 (32.4)	0.252
Hypertension, *n* (%)	154 (87.0)	77 (85.5)	45 (90.0)	32 (86.5)	0.751
Diastolic pressure (mmHg)	84 ± 15	81 ± 14	88 ± 16	83 ± 17	0.149
Systolic pressure (mmHg)	143 ± 27	138 ± 23	152 ± 34	142 ± 22	0.069
Alcohol consumption, *n* (%)	52 (29.5)	31 (34.8)	11 (22.0)	10 (27.0)	0.263
Cigarette smoking, *n* (%)	41 (23.2)	16 (23.5)	13 (30.2)	8 (24.2)	0.551
Physical activity, *n* (%)	65 (36.9)	39 (43.3)	16 (32.0)	10 (27.8)	0.183
Glucose (mmol/L)	6.6 ± 3.2	5.7 ± 2.1	7.9 ± 4.5	6.7 ± 3.1	0.001^b,c^
Total cholesterol (mmol/L)	4.6 ± 1.3	4.6 ± 1.1	4.7 ± 1.3	4.8 ± 1.6	0.648
HDL cholesterol (mmol/L)	1.0 ± 0.3	0.9 ± 0.3	0.9 ± 0.3	0.9 ± 0.3	0.751
LDL cholesterol (mmol/L)	2.8 ± 1.1	2.7 ± 1.0	2.8 ± 1.1	3.1 ± 1.4	0.248
Triglycerides (mmol/L)	1.9 ± 1.2	1.9 ± 1.2	1.9 ± 1.4	1.7 ± 0.6	0.875
ALT (*µ*kat/L)	0.5 ± 0.3	0.5 ± 0.3	0.5 ± 0.4	0.5 ± 0.3	0.639
AST (*µ*kat/L)	0.5 ± 0.3	0.6 ± 0.3	0.5 ± 0.3	1.8 ± 1.0	0.506
Urea (mmol/L)	6.4 ± 2.0	6.2 ± 1.8	6.3 ± 1.9	6.6 ± 2.6	0.831
Creatinine (*μ*mol/L)	82.1 ± 23.1	82.5 ± 22.2	83.0 ± 23.7	79.6 ± 25.0	0.758

Categorical variables are shown as number of patients (percentage) and compared using the chi-square test. Continuous variables are presented as mean ± standard deviation and compared using one-way ANOVA followed by Tukey's test for multiples comparisons; *p* < 0.05 was considered statistically significant. CAD: coronary artery disease; BMI: body mass index; HDL: high-density lipoprotein; LDL: low-density lipoprotein; AST: aspartate aminotransferase; ALT: aminotransferase. ^a^*p*=0.05 for comparison between 0–4 and 10–15 Friesinger index groups. ^b^*p* < 0.001 for comparison between 0–4 and 5–9 Friesinger index groups. ^c^*p*=0.015 for comparison between 0–4 and 10–15 Friesinger index groups.

**Table 2:** Serum alpha-tocopherol and retinol levels in patients with coronary artery disease.

Variables	Extent of coronary lesion (Friesinger index)			
	0–4	5–9	10–15	*p* value
*Alpha-tocopherol*	(*n*=42)	(*n*=23)	(*n*=17)	
** ** *μ*mol/mmol cholesterol	4.4 ± 2.4	6.2 ± 3.7	6.3±2.8	0.036^a^
** ** *μ*mol/L	19.3 ± 11.6	26.8 ± 12.8	27.1 ± 15.2	0.045^a^
** **Adequate levels, *n* (%)	28 (66.7)	20(87.0)	16 (94.1)	0.033
** **Low levels, *n* (%)	14 (33.3)	3 (13.0)	1 (5.9)	
*Retinol*	(*n*=65)	(*n*=50)	(*n*=26)	
** ** *μ*mol/L	1.91 ± 0.77	2.03 ± 0.80	2.17 ± 1.0	0.492
** **Adequate levels, *n* (%)	60 (92.3)	34 (94.4)	26 (100.0)	0.348
** **Low levels, *n* (%)	5 (7.7)	2 (5.6)	0 (0)	

Categorical variables are shown as the number of patients (percentage) and compared using the chi-square test. Continuous variables are shown as mean ± standard deviation and compared using one-way ANOVA followed by Tukey's test for multiples comparisons; *p* < 0.05 was considered statistically significant. CAD: coronary artery disease. ^a^*p* < 0.05 for comparison between 0–4 and 5–9 Friesinger index groups.

**Table 3:** Multiple logistic regression (dependent variable Friesinger index).

Variables	B	OR (95% CI)	*p* value
Glucose (mmol/L)	0.005	1.0 (1.0–1.02)	0.347
Age (years)	0.112	1.1 (1.0–1.21)	0.008
Alpha-tocopherol (*μ*mol/L)	2.443	11.5 (1.2–112.1)	0.035

Dependent variable: Friesinger index (0: FI 0–4; 1: FI 10–15). Independent variables: age and glucose (continuous variables) and serum alpha-tocopherol levels (categorical variable) (0: <12 *μ*mol/L; 1:>12 *μ*mol/L). OR, odds ratio; CI, confidence interval.

## Data Availability

The data used to support the findings of this study are included within the article.

## References

[1] Grober E. D., Bohnen J. M. A. (2015). Global, regional, and national age–sex specific all-cause and cause-specific mortality for 240 causes of death, 1990–2013: a systematic analysis for the global burden of disease study 2013. *The Lancet*.

[2] Rosen M., Chan P., Saleem M. (2018). Longitudinal associations between 4-hydroxynonenal and depression in coronary artery disease patients. *Psychiatry Research*.

[3] Zhang L., Zhang Y., Zhao Y. (2018). Circulating miRNAs as biomarkers for early diagnosis of coronary artery disease. *Expert Opinion on Therapeutic Patents*.

[4] Frostegård J. (2013). Immunity, atherosclerosis and cardiovascular disease. *BMC Medicine*.

[5] Husain K., Hernandez W., Ansari R. A., Ferder L. (2015). Inflammation, oxidative stress and renin angiotensin system in atherosclerosis. *World Journal of Biological Chemistry*.

[6] Marchioli R. (1999). Antioxidant vitamins and prevention of cardiovascular disease: laboratory, epidemiological and clinical trial data. *Pharmacological Research*.

[7] Brazionis L., Walker K. Z., Itsiopoulos C., O’Dea K. (2012). Plasma retinol: a novel marker for cardiovascular disease mortality in Australian adults. *Nutrition, Metabolism and Cardiovascular Diseases*.

[8] Lonn E., Bosch J., Yusuf S. (2005 Mar 16). Effects of long-term vitamin E supplementation on cardiovascular events and cancer. *JAMA*.

[9] Adams A. K., Wermuth E. O., McBride P. E. (1999). Antioxidant vitamins and the prevention of coronary heart disease. *American Family Physician*.

[10] Rashidi B., Hoseini Z., Sahebkar A., Mirzaei H. (2016). Anti-atherosclerotic effects of vitamins D and E in suppression of atherogenesis. *Journal of cellular physiology*.

[11] Mathur P., Ding Z., Saldeen T., Mehta J. L. (2015). Tocopherols in the prevention and treatment of atherosclerosis and related cardiovascular disease. *Clinical Cardiology*.

[12] Costacou T., Evans R. W., Schafer G. L., Orchard T. J. (2013). Oxidative stress and response in relation to coronary artery disease in type 1 diabetes. *Diabetes Care*.

[13] Guimarães M. R. M., Murad L. B., Paganelli A., de Oliveira C. A. B., Vianna L. M. A. (2015). Effects of alpha-tocopherol associated with lovastatin on brain tissue and memory function in SHRSPs. *Physiology & Behavior*.

[14] Albahrani A. A., Greaves R. F. (2016). Fat-soluble vitamins : clinical indications and current challenges for chromatographic measurement. *Clinical Biochemistry Reviews*.

[15] Ragino Y. I., Chernyavski A. M., Polonskaya Y. V., Volkov A. M., Semaeva E. V., Voevoda M. I. (2007). Oxidative and antioxidant changes during formation of unstable atherosclerotic plaque. *Bulletin of Experimental Biology and Medicine*.

[16] Min K.-B., Min J.-Y. (2014). Relation of serum vitamin A levels to all-cause and cause-specific mortality among older adults in the NHANES III population. *Nutrition, Metabolism and Cardiovascular Diseases*.

[17] De Keyser J., De Klippel N., Merkx H., Vervaeck M., Herroelen L. (1992). Serum concentrations of vitamins A and E and early outcome after ischaemic stroke. *The Lancet*.

[18] Duarte M. K. R. N., de Araújo J. N. G., Duarte V. H. R. (2016). The relationship of the oleic acid level and ECHDC3 mRNA expression with the extent of coronary lesion. *Lipids in Health and Disease*.

[19] Duarte V. H. R. Avaliação dos polimorfismos e expressão de RNAm do TREML4 em leucócitos de pacientes com doença arterial coronariana.

[20] Chagas P., Caramori P., Galdino T. P., Barcellos C. D. S. D., Gomes I., Schwanke C. H. A. (2013). Egg consumption and coronary atherosclerotic burden. *Atherosclerosis*.

[21] Friedewald W. T., Levy R. I., Fredrickson D. S. (1972). Estimation of the concentration of low-density lipoprotein cholesterol in plasma, without use of the preparative ultracentrifuge. *Clinical Chemistry*.

[22] Ortega R. M., López-Sobaler A. M., Elena Quintas M., Martínez R. M., Andrés P. (1998). The influence of smoking on vitamin C status during the third trimester of pregnancy and on vitamin C levels in maternal milk. *Journal of the American College of Nutrition*.

[23] da Silva Ribeiro K. D., Lima M. S. R., Medeiros J. F. P. (2016). Association between maternal vitamin E status and alpha-tocopherol levels in the newborn and colostrum. *Maternal & Child Nutrition*.

[24] Nierenberg D. W., Nann S. L. (1992). A method for determining concentrations of retinol, tocopherol, and five carotenoids in human plasma and tissue samples. *American Journal of Clinical Nutrition*.

[25] Milne D. B., Botnen J. (1986). Retinol, alpha-tocopherol, lycopene, and alpha- and beta-carotene simultaneously determined in plasma by isocratic liquid chromatography. *Clin Chem*.

[26] IOM (2000). *Dietary Reference Intakes for Vitamin C, Vitamin E, Selenium and Carotenoids*.

[27] WHO (2009). *Global Prevalence of Vitamin A Deficiency in Populations at Risk 1995–2005, WHO Global Database on Vitamin A Deficiency*.

[28] Faul F., Erdfelder E., Lang A.-G., Buchner A. (2007). G^∗^Power 3: a flexible statistical power analysis program for the social, behavioral, and biomedical sciences. *Behavior Research Methods*.

[29] Li G., Li Y., Chen X., Sun H., Hou X., Shi J. (2015). Circulating tocopherols and risk of coronary artery disease: a systematic review and meta-analysis. *European Journal of Preventive Cardiology*.

[30] Apostolidou C., Adamopoulos K., Lymperaki E., Iliadis S., Papapreponis P., Kourtidou-Papadeli C. (2015). Cardiovascular risk and benefits from antioxidant dietary intervention with red wine in asymptomatic hypercholesterolemics. *Clinical Nutrition ESPEN*.

[31] Jayedi A., Rashidy-Pour A., Parohan M., Sadat Zargar M., Shab-Bidar S. (2018). Dietary antioxidants, circulating antioxidant concentrations, total antioxidant capacity, and risk of all-cause mortality: a systematic review and dose-response meta-analysis of prospective observational studies. *Advances in Nutrition*.

[32] Jenab M., Riboli E., Ferrari P. (2006). Plasma and dietary vitamin C levels and risk of gastric cancer in the european prospective investigation into cancer and nutrition (EPIC-EURGAST). *Carcinogenesis*.

[33] Luo H., Fang Y.-J., Lu M.-S. (2018). Dietary and serum vitamins A and E and colorectal cancer risk in Chinese population. *European Journal of Cancer Prevention*.

[34] Susan T. M. (2003). Antioxidant nutrients and chronic disease: use of biomarkers of exposure and oxidative stress status in epidemiologic research. *Journal of Nutrition*.

[35] Ford E. S., Sowell A. (1999). Serun-tecopherol status in the United States population: findings from the third national health and nutrition examination survey. *American Journal of Epidemiology*.

[36] Mah E., Sapper T. N., Chitchumroonchokchai C. (2015). -Tocopherol bioavailability is lower in adults with metabolic syndrome regardless of dairy fat co-ingestion: a randomized, double-blind, crossover trial. *American Journal of Clinical Nutrition*.

[37] Tanumihardjo S. A., Russell R. M., Stephensen C. B. (2016). Biomarkers of nutrition for development (BOND)-vitamin A review. *Journal of Nutrition*.

[38] Borel P., Desmarchelier C. (2017). Genetic variations associated with vitamin a status and vitamin A bioavailability. *Nutrients*.

[39] Singh N., Singh N., Singh S. K., Singh A. K., kafle D., Agrawal N. (2012). Reduced antioxidant potential of LDL is associated with increased susceptibility to LDL peroxidation in type II diabetic patients. *International Journal of Endocrinology and Metabolism*.

[40] Traber M. G. (2013). Mechanisms for the prevention of vitamin E excess. *Journal of Lipid Research*.

[41] Upston J. M., Terentis A. C., Stocker R. (1999). Tocopherol-mediated peroxidation of lipoproteins: implications for vitamin E as a potential antiatherogenic supplement. *FASEB Journal*.

[42] Nagao M., Moriyama Y., Yamagishi K., Iso H., Tamakoshi A. (2012). Relation of serum ^|^alpha;- and ^|^gamma;-Tocopherol levels to cardiovascular disease-related mortality among Japanese men and women. *Journal of Epidemiology*.

[43] Polidori C. M., Praticó D., Parente B. (2007). Elevated lipid peroxidation biomarkers and low antioxidant status in atherosclerotic patients with increased carotid or iliofemoral intima media thickness. *Journal of Investigative Medicine*.

